# Supplementary material for: Linear response theory of open systems with exceptional points
Source: Nat Commun. 2022 Jun 7;13:3281. doi: 10.1038/s41467-022-30715-8 (PMC9174331; doi:10.1038/s41467-022-30715-8)
Supplement: Supplementary file 1 — Supplementary information [file 41467_2022_30715_MOESM1_ESM.pdf]

Supplementary Information: Linear response theory of open systems with exceptional points

A. Hashemi, K. Busch, D. N. Christodoulides, S.K. Ozdemir, and R. El-Ganainy

SUPPLEMENTARY NOTES

1. Subtleties arising in resonant systems with EPs: an example

Here we provide a detailed analysis for the example shown in Fig. 2 in the main text, which is also shown here for convenience. It consists of three identical microring resonators that are coupled sequentially via horizontal waveguides. An additional vertical waveguide provides access to selectively excite the second resonator. We neglect the cross talk between the horizontal and vertical waveguides since it can be minimized using various design strategies [1–4]. A similar system was considered in [5] and shown to exhibit a third order EP in the subspace spanned by the CW, CCW and CW modes of the resonators $R_{1,2,3}$, respectively. In this subspace, and by allowing excitations only from ports $P_{1,2,3}$ with the collection output ports Q_1 and Q_2 (see supplementary Fig. 1) the system is described by the coupled equations:

$$i\frac{d}{dt}\begin{bmatrix} a_1 \\ a_2 \\ a_3 \end{bmatrix} = \begin{bmatrix} \omega_o - 3i\gamma & 0 & 0 \\ \kappa & \omega_o - 3i\gamma & 0 \\ 0 & \kappa & \omega_o - 3i\gamma \end{bmatrix} \begin{bmatrix} a_1 \\ a_2 \\ a_3 \end{bmatrix} + i\sqrt{2\gamma}\begin{bmatrix} P_1 \\ P_2 \\ P_3 \end{bmatrix},$$

$$Q_1 = P_3 - \sqrt{2\gamma}\, a_3,$$

$$Q_2 = P_I - \sqrt{2\gamma}\, a_3, \tag{1.1}$$

where $a_{1,2,3}$ are modal amplitudes of the CW, CCW and CW modes associated with resonators $R_{1,2,3}$, respectively. The resonant frequency and decay rate of each resonator are given by ω_o and 3γ. For resonator R_2 the decay rate is due to equal coupling to three waveguides. On the-other hand, for $R_{1,3}$ the coupling to waveguides contributes only 2γ and we assume that an additional loss of γ is intentionally introduced for example by depositing a metal layer on top of the ring or introducing an auxiliary waveguide next to each existing waveguide. The ports $P_{1,2,3}$ serve as excitation channels for the modes $a_{1,2,3}$, respectively, while the output signal is collected from ports Q_1 and Q_2 as depicted in supplementary Fig. 1. In addition, P_I represents the signal in the waveguide between R_2 and R_3 (see supplementary Fig. 1**b**). Within this modal subspace, there is unidirectional coupling from modes $a_1 \rightarrow a_2$ and $a_2 \rightarrow a_3$. The exact details of this waveguide-mediated indirect coupling depends on the evanescent tunneling between the rings and waveguides as well as the

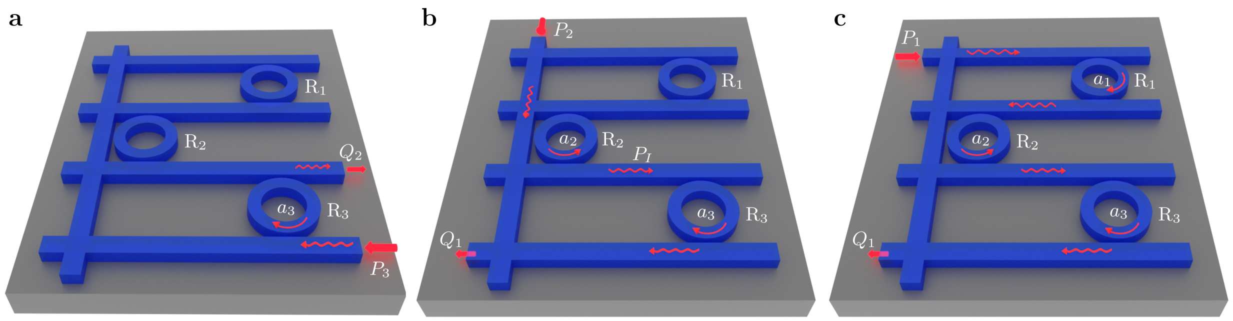

Supplementary Fig. 1. **Subtleties of linear response of non-Hermitian systems having EPs: an illustrative example.** A photonic system that exhibits an EP of order three. As described in the main text, it can have very different linear responses based on the input/output channel configuration. Here we present the detailed mathematical analysis of this structure.

distances between the rings. Here we assume that unidirectional coupling coefficients are identical and denote them by κ.

In this case, the resolvent, G_3, can be evaluated in closed form:

$$G_3 = \begin{bmatrix} \mu_3 & 0 & 0 \\ \kappa\mu_3^2 & \mu_3 & 0 \\ \kappa^2\mu_3^3 & \kappa\mu_3^2 & \mu_3 \end{bmatrix}, \tag{1.2}$$

where $\mu_3 \equiv 1/(\omega - \omega_o + 3i\gamma)$. One can easily confirm that the exceptional eigenvector of G_3 is $|K_1^r\rangle = [0, 0, 1]^T$, while the generalized eigenvectors forming the Jordan chain are give by $|K_2^r\rangle = [0, \frac{\xi}{\kappa\mu_3^2}, 0]^T$ and $|K_3^r\rangle = \left[(\frac{\xi}{\kappa\mu_3^2})^2, -\frac{\chi_o^2}{\kappa\mu_3^3}, 0\right]^T$ (see Eq.(5.1) of supplementary note 6).

Next, we consider the above system when it is excited from one of the ports $P_{1,2,3}$. These signal pathways inside the structure for each of these distinct situations are depicted in supplementary Figs. 1**a**, **b**, and **c**. It is straightforward to show that the scattering coefficient between the input and output ports in each case is given by:

$$\begin{aligned} \frac{Q_2}{P_3} &= -2i\gamma\mu_3 = -\frac{2i\gamma}{(\omega - \omega_o + 3i\gamma)}, \\ \frac{Q_1}{P_2} &= -2i\gamma\kappa\mu_3^2 = -\frac{2i\gamma\kappa}{(\omega - \omega_o + 3i\gamma)^2}, \\ \frac{Q_1}{P_1} &= -2i\gamma\kappa^2\mu_3^3 = -\frac{2i\gamma\kappa^2}{(\omega - \omega_o + 3i\gamma)^3}. \end{aligned} \tag{1.3}$$

These results can be intuitively understood by referring again to supplementary Fig. 1, which depicts the designated input and output ports, and the actual paths taken by the excitation signal. In the first case, the input signal P_3 crosses only the ring resonator R_3 before it couples to the output Q_2. On the other hand, the signal P_2 in the second case interacts with two rings (R_2 and R_3) before it is coupled to the output channel Q_1. Finally, the trajectory of the input signal P_1 in the third case involves three resonators and the output port Q_1. The results discussed above can be understood by noting that each ring contributes a Lorentzian response and that total response is the product of the individual responses (due to the series connection of the rings). In more complex systems where such a simple trajectory picture does not exist, it is necessary to develop a general theoretical framework to analyze and understand the system's response.

2. Completeness and biorthogonality of the eigenvectors associated with defective matrices

The eigenvectors of non-Hermitian matrices are in general non-orthogonal. Instead, they obey a modified bi-orthogonality relation, i.e. orthogonality between the right eigenvector and its corresponding left eigenvectors. The spectral analysis of a non-Hermitian matrix becomes rather complicated when it is defective, i.e. exhibiting EPs in its spectrum. In what follows, we provide a concise discussion that elaborates on this situation. Let us assume an $N \times N$ matrix $\hat{H}_{EP}$ that has one EP of order M (generalization to multiple EPs is straightforward). The right eigenvectors ($|\psi_n^r\rangle$) of $\hat{H}_{EP}$ are defined by

$$(\hat{H}_{EP} - \Omega_n \hat{I}) \, |\psi_n^r\rangle = 0, \tag{2.1}$$

where $n = 1, 2, \dots, N-M$. Evidently, these vectors do not span the underlying vector space. It is well-known however that the bases can be completed by using the Jordan canonical chain procedure, i.e. by defining the generalized right eigenvectors ($|J_m^r\rangle$) of $\hat{H}_{EP}$ by the Jordan chain

$$(\hat{H}_{EP} - \Omega_{EP} \hat{I}) \, |J_m^r\rangle = \chi_m \, |J_{m-1}^r\rangle \,, \tag{2.2}$$

where $m = 1, 2, \dots, M$ and $\chi_1 = 0$. The eigenvectors $|J_m^r\rangle$ are linearly independent. This can be shown as follows. Consider the relation $\sum_{m=1}^{M} z_m \, |J_m^r\rangle = 0$. We now apply the operator

$(H - \Omega_{EP}\hat{I})^{M-1}$ to both sides. All the terms on the left-hand-side will vanish except the last term, which gives $z_M = 0$. By repeating this procedure on the remaining terms, we find that all the coefficients z_m have to be zero. Similarly, one can show that any vector $|J^r_m\rangle$ is linearly independent from all the vectors $|\psi^r_n\rangle$. In other words, the set $\{|\psi^r_n\rangle\}$ spans an $N - M$ dimensional space (call it $\mathcal{D}_\phi$) while the set $\{|J^r_n\rangle\}$ spans the complementary M dimensional space $\mathcal{D}_{EP}$. Taken together, the set $\{|\psi^r_n\rangle, |J^r_n\rangle\}$ forms a complete basis that spans $\mathcal{D}_\phi \cup \mathcal{D}_{EP}$. Thus any vector in that space can be decomposed in terms of these bases. However, as we mentioned, in general $\langle\psi^r_m|\psi^r_n\rangle \neq \delta_{m,n}$. This, in turn complicates the procedure for finding the projection of any general vector onto the bases vectors. This difficulty can be overcome by using the concept of left eigenvectors:

$$\langle\psi^l_n| (\hat{H}_{EP} - \Omega_n\hat{I}) = 0. \tag{2.3}$$

The set $\{\langle\psi^l_m|\}$ defines a dual space of $\mathcal{D}_\phi$ (see Fig. 5**a**). From Eq. (2.1), we can write $\langle\psi^l_{n_2}|\hat{H}_{EP}|\psi^r_{n_1}\rangle = \Omega_{n_1}\langle\psi^l_{n_2}|\psi^r_{n_1}\rangle$. Similarly, from Eq. (2.3) we obtain $\langle\psi^l_{n_2}|\hat{H}_{EP}|\psi^r_{n_1}\rangle = \Omega_{n_2}\langle\psi^l_{n_2}|\psi^r_{n_1}\rangle$. For these two relations to be consistent for $\Omega_{n_1} \neq \Omega_{n_2}$, we must have $\langle\psi^l_{n_2}|\psi^r_{n_1}\rangle = 0$ for $n_1 \neq n_2$. This is known as biorthogonality. By using this last relation iteratively in the recursive system of Eqs. (2.2), we also find that $\langle\psi^l_m|J^r_n\rangle = 0$ for any m and n. For instance, by using $(\hat{H}_{EP} - \Omega_{EP}\hat{I})^2|J^r_2\rangle = 0$, we find that $\langle\psi^l_m|(\hat{H}_{EP} - \Omega_{EP}\hat{I})^2|J^r_2\rangle = 0$. On the other hand, by using $\langle\psi^l_m|(\hat{H}_{EP} - \Omega_{EP}\hat{I})^2 = \langle\psi^l_m|(\Omega_m - \Omega_{EP})^2$, the same expression can be evaluated to be $\langle\psi^l_m|(\hat{H}_{EP} - \Omega_{EP}\hat{I})^2|J^r_2\rangle = (\Omega_m - \Omega_{EP})^2\langle\psi^l_m|J^r_2\rangle$, which gives $\langle\psi^l_m|J^r_2\rangle = 0$.

Similarly, one can define the left generalized eigenvectors of $\hat{H}_{EP}$ by following the same procedure, namely:

$$\langle J^l_m| (\hat{H}_{EP} - \Omega_{EP}\hat{I}) = \chi_m \langle J^l_{m-1}|. \tag{2.4}$$

Following the same procedure as before, one can show that the vectors $\langle J^l_m|$ are linearly independent from each other and from $\langle\psi^l_n|$, i.e. they define a dual space of $\mathcal{D}_{EP}$ (see Fig. 5**a** in the main text). Also, it is straightforward to show that $\langle J^l_m|\psi^r_n\rangle = 0$ for any m and n. On the other hand, the relation between the vectors $\langle J^l|$ and $|J^r\rangle$ is subtle due to the self-orthogonality: $\langle J^l_m|J^r_m\rangle = 0$ for $m \leq \frac{M}{2}$. This can be proven by considering the expression $\langle J^l_m|(\hat{H}_{EP} - \Omega_{EP}\hat{I})^m|J^r_{2m}\rangle$. When evaluated by first calculating the term

$\langle J^l_m| (\hat{H}_{EP} - \Omega_{EP}\hat{I})^m$, we find that the result is zero. On the other hand, when we first evaluate the term $(\hat{H}_{EP}-\Omega_{EP}\hat{I})^m |J^r_{2m}\rangle$, we obtain $\chi_{2m}\chi_{2m-1}\ldots\chi_{m+1} \langle J^l_m|J^r_m\rangle$, which proves the self-orthogonality relation.

In order to overcome the above problem, we first note that the set of the left eigenvectors $\{\langle J^l_m|\}$ spans the dual space of $\mathcal{D}_{EP}$. Thus any vector formed by an arbitrary superposition of $\{\langle J^l_m|\}$ also lies in the dual space of $\mathcal{D}_{EP}$. Let us now define a new set of vectors $\langle \tilde{J}^l_m|$, each of which lies in the dual space of $\mathcal{D}_{EP}$, i.e. $\langle \tilde{J}^l_m| = \sum_{k=1}^{M} t_{m,k} \langle J^l_k|$. In order to find the M^2 coefficients $t_{m,k}$, we need to impose M^2 normalization conditions. These can be chosen to satisfy $\langle \tilde{J}^l_m|J^r_k\rangle = \delta_{m,k}$. Note that the new vectors $\langle \tilde{J}^l_m|$ are not generalized eigenvectors of the Hamiltonian $\hat{H}_{EP}$.

3. Simultaneous normalization of right and left eigenvectors

The simultaneous normalization of the right and left eigenvectors associated with a non-Hermitian Hamiltonian was discussed in [6]. For completeness, we reiterate some of these results here from a more general point of view. The main result of this section is that for non-normal matrices (matrices that do not commute with their Hermitian conjugates), the three different normalization conditions $\langle\psi^l_n|\psi^r_n\rangle = 1$, $\langle\psi^r_n|\psi^r_n\rangle = 1$, and $\langle\psi^l_n|\psi^l_n\rangle = 1$ cannot be satisfied simultaneously. For non-defective Hamiltonians, this is a direct consequence of Cauchy-Schwarz inequality: $|\langle\psi^l_n|\psi^r_n\rangle|^2 \leq \langle\psi^r_n|\psi^r_n\rangle \langle\psi^l_n|\psi^l_n\rangle$. The equality holds if and only if the vectors $|\psi^r_n\rangle$ and $|\psi^l_n\rangle$ are the same up to a constant. This occurs when $\hat{H}$ is normal, i.e. $[\hat{H}, \hat{H}^\dagger] = 0$. To prove this, we note that the above relation implies that both $\hat{H}$ and $\hat{H}^\dagger$ share the same right and left eigenvectors. It follows that $|\psi^r_n\rangle$ and $\langle\psi^l_n|$ are also right and left eigenvectors of the operator $\hat{H}\hat{H}^\dagger$. This last operator is Hermitian and thus, up to a constant, the right and left eigenvectors are identical.

4. Series expansion of the resolvent

In this section, we present a detailed derivation for the series expansion of the resolvent in Eq. (6). By considering an arbitrary input signal $|u\rangle = \sum_{n=1}^{N-M} c_n |\psi^r_n\rangle + \sum_{m=1}^{M} d_m |J^r_m\rangle$,

where the coefficients c_n and d_m are known (or can be calculated using the projection rules for the right/left eigenvectors as discussed in the previous section), and by assuming a similar series representation of the response signal with known coefficients $\tilde{c}_n$ and $\tilde{d}_m$, we can write:

$$(\omega\hat{I}-\hat{H}_{EP})\left(\sum_{n=1}^{N-M}\tilde{c}_n\left|\psi_n^r\right\rangle+\sum_{m=1}^{M}\tilde{d}_m\left|J_m^r\right\rangle\right)=\sum_{n=1}^{N-M}c_n\left|\psi_n^r\right\rangle+\sum_{m=1}^{M}d_m\left|J_m^r\right\rangle. \tag{4.1}$$

By using Eqs. (2.1) and (2.2), we can express the left-hand-side of Eq. (4.1) as $\sum_{n=1}^{N-M}\tilde{c}_n(\omega-\Omega_n)\left|\psi_n^r\right\rangle+\sum_{m=1}^{M}\left[\tilde{d}_m(\omega-\Omega_{EP})-\tilde{d}_{m+1}\chi_{m+1}\right]\left|J_m^r\right\rangle$. By recalling that the vectors $\left|\psi_n^r\right\rangle$ and $\left|J_m^r\right\rangle$ are all linearly independent, we arrive at:

$$\tilde{c}_n=\frac{c_n}{\omega-\Omega_n} \tag{4.2}$$

$$\tilde{d}_m=\frac{d_m}{\omega-\Omega_{EP}}+\frac{\chi_{m+1}\tilde{d}_{m+1}}{\omega-\Omega_{EP}}. \tag{4.3}$$

The recursive relation in Eq. (4.3) can be further simplified by noting that $\chi_{M+1}=0$, which gives $\tilde{d}_M=\frac{d_M}{\omega-\Omega_{EP}}$. By inserting this result back in Eq. (4.3), we obtain the general expression:

$$\begin{aligned}\tilde{d}_m=&\frac{d_m}{\omega-\Omega_{EP}}+\frac{\chi_{m+1}d_{m+1}}{(\omega-\Omega_{EP})^2}+\frac{\chi_{m+1}\chi_{m+2}d_{m+2}}{(\omega-\Omega_{EP})^3}+\cdots+\frac{\chi_{m+1}\chi_{m+2}\cdots\chi_M d_M}{(\omega-\Omega_{EP})^{M-m+1}}\\=&\sum_{k=m}^{M}\alpha_k^{(m)}\frac{d_k}{(\omega-\Omega_{EP})^{k-m+1}},\end{aligned} \tag{4.4}$$

where we defined $\alpha_m^{(m)}=1$ and $\alpha_k^{(m)}=\alpha_{k-1}^{(m)}\chi_k=\chi_k\chi_{k-1}\dots\chi_{m+1}$ for $k=m+1,\dots,M$. Finally, by using $c_n=\left\langle\psi_n^l|u\right\rangle$, $d_k=\langle\tilde{J}_k^l|u\rangle$ (see supplementary note 2) we obtain:

$$\hat{G}_{EP}\left|u\right\rangle=\left(\sum_{n=1}^{N-M}\frac{\left|\psi_n^r\right\rangle\left\langle\psi_n^l\right|}{\omega-\Omega_n}+\sum_{m=1}^{M}\sum_{k=m}^{M}\alpha_k^{(m)}\frac{\left|J_m^r\right\rangle\langle\tilde{J}_k^l|}{(\omega-\Omega_{EP})^{k-m+1}}\right)\left|u\right\rangle. \tag{4.5}$$

Since $\left|u\right\rangle$ is an arbitrary vector, it follows that the series summation on the right-hand-side represents the resolvent which proves Eq. (6).

5. Non-interfering excitation channels

Here we discuss the classification of the excitation channels based on their interference properties. Consider an input $\left|u\right\rangle=\left|u_1\right\rangle+\left|u_2\right\rangle$, with $s(t)=e^{-i\omega_e t}$. This rather unphysical assumption of an input signal that extends over the time axis is well suited for treating

long time limits after the transient response has died out, which is the case of interest here (alternatively, one can, of course, use the Laplace transform and take the long-time limit $t \to \infty$ explicitly). The normalized energy (with respect to some reference energy value), stored in the system after the transient response fades away, is given by $\langle E\rangle \equiv \lim_{t\to\infty} \overline{\langle a(t)|a(t)\rangle}$, with the overline indicating time average. Equivalently, the normalized stored energy can be also expressed as $\langle E\rangle = \langle \tilde{A}(\omega_e)|\tilde{A}(\omega_e)\rangle$ where $|A(\omega)\rangle = |\tilde{A}(\omega_e)\rangle\, \delta(\omega-\omega_e)$, or $|\tilde{A}(\omega_e)\rangle = \sum_{n=1}^{N} \frac{c_n}{\omega_e-\Omega_n} |\psi_n^r\rangle$. In general, the above expression will contain contributions from (1) $|u_1\rangle$ only in the absence of $|u_2\rangle$; (2) $|u_2\rangle$ only in the absence of $|u_1\rangle$; (3) interference component due to the non-orthogonality of $|u_{1,2}\rangle$. To illustrate this with a concrete example, consider an input $|f\rangle = [\alpha_m |\psi_m^r\rangle + \alpha_n |\psi_n^r\rangle]\, e^{-i\omega_e t}$. The normalized stored energy in this case is given by $\langle E\rangle = |\frac{\alpha_m}{\omega_e-\Omega_m}|^2 \langle\psi_m^r|\psi_m^r\rangle + |\frac{\alpha_n}{\omega_e-\Omega_n}|^2 \langle\psi_n^r|\psi_n^r\rangle + 2\mathrm{Re}\{\frac{\alpha_m^*}{\omega_e-\Omega_m^*}\frac{\alpha_n}{\omega_e-\Omega_n}\langle\psi_m^r|\psi_n^r\rangle\}$), with the last term representing the interference term. This raises the question of whether this feature is pertinent to any input profile or if it is possible to construct some excitation channels whose energy contribution inside the resonators do not interfere. The main result of this section indeed affirms the latter possibility. To demonstrate this, we first note that the resolvent $\hat{G}_{EP}(\omega)$ has the same spectral structure of the Hamiltonian $\hat{H}_{EP}$, i.e. they have the same eigenvectors and their eigenvalues are given by $\frac{1}{\omega-\Omega_n}$ and Ω_n respectively. Consequently, they also share the same EP (see supplementary note 5). As a result, one can in principle complete the basis by constructing the Jordan vectors associated with $\hat{G}_{EP}(\omega)$ instead of $\hat{H}_{EP}$ as we have done before:

$$\begin{aligned}
(\hat{G}_{EP} - \mu_{EP}\hat{I})\,|K_1^r\rangle &= 0\\
(\hat{G}_{EP} - \mu_{EP}\hat{I})\,|K_2^r\rangle &= \xi\,|K_1^r\rangle\\
&\vdots\\
(\hat{G}_{EP} - \mu_{EP}\hat{I})\,|K_M^r\rangle &= \xi\,|K_{M-1}^r\rangle\,.
\end{aligned} \tag{5.1}$$

In the above, $\mu_{EP} = \frac{1}{\omega-\Omega_{EP}}$ and the coefficient ξ is taken to be of unit value and of dimensions similar to μ_{EP}. We emphasize that both $\hat{G}_{EP}$ and μ_{EP} are functions of ω. It is straightforward to show that $|K_1^r\rangle = |J_1^r\rangle$. A more general Jordan vector $|K_m^r\rangle$ with $m > 1$, can be expressed as a linear superposition of the vectors $|J_m^r\rangle$ (see supplementary note 6). We now consider the case when the matrix representation of $\hat{G}_{EP}$ is given by the Jordan canonical form. In this case, the generalized eigenvectors are orthogonal. This is of course a feature of the geometry of the problem. For instance, if the effective

Hamiltonian of the optical structure under study is given by $\hat{H} = \begin{pmatrix} \Omega & J & \kappa \\ 0 & \Omega & J \\ 0 & 0 & \Omega \end{pmatrix}$, then we find $\hat{G}(\omega = \Omega - \frac{J^2}{\kappa}) = \frac{\kappa^2}{J^3} \begin{pmatrix} -\frac{J}{\kappa} & 1 & 0 \\ 0 & -\frac{J}{\kappa} & 1 \\ 0 & 0 & -\frac{J}{\kappa} \end{pmatrix}$ with $|K_1^r\rangle = [1,0,0]^T$, $|K_2^r\rangle = [0,1,0]^T$, and $|K_3^r\rangle = [0,0,1]^T$. By assuming that the above condition is satisfied, we now consider an input of the form $|f\rangle = [\alpha_m |K_m^r\rangle + \alpha_n |K_n^r\rangle] e^{-i\omega_e t}$. Clearly, if $|n-m| = 1$, interference terms will appear in the expression for $\langle E \rangle$. On the other hand, when $|n-m| > 1$, the energy expression will not contain any interference terms. For instance, for $m = 2$ and $n = 4$, we will obtain $\langle E \rangle = \langle E_2 \rangle + \langle E_4 \rangle$, where $\langle E_2 \rangle = |\alpha_2|^2 [|\xi|^2 \langle K_1^r | K_1^r \rangle + |\mu_{EP}(\omega_e)|^2 \langle K_2^r | K_2^r \rangle]$ and $\langle E_4 \rangle = |\alpha_4|^2 [|\xi|^2 \langle K_3^r | K_3^r \rangle + |\mu_{EP}(\omega_e)|^2 \langle K_4^r | K_4^r \rangle]$. If, on the other hand, the natural matrix representation (i.e. the representation that arises from the geometry of the problem without performing any linear mapping) of $\hat{G}$ is not in the Jordan canonical form, the generalized eigenvectors do not have to be orthogonal. However, by using the similarity transformation that relates the natural and canonical bases one can find the non-interfering channels in the natural bases. These in general will be a superposition between the excitation channels associated with the natural bases.

The above analysis in terms of the Jordan vectors associated with the resolvent illustrates another interesting effect. Consider the two different inputs $|f_{1,2}\rangle = \alpha_{1,2} |K_{1,2}^r\rangle e^{-i\omega_e t}$, with the constants $\alpha_{1,2}$ chosen to satisfy the conditions $|\alpha_{1,2}|^2 \langle K_{1,2}^r | K_{1,2}^r \rangle = 1$. In this case, the expressions for the corresponding energy stored in the structure are given by: $\langle E_1 \rangle = |\mu_{EP}(\omega_e)|^2$, and $\langle E_2 \rangle = |\mu_{EP}(\omega_e))|^2 + |\xi|^2 |\alpha_2/\alpha_1|^2$. In other words, $\langle E_2 \rangle / \langle E_1 \rangle = 1 + \frac{\xi^2}{|\mu_{EP}|^2} |\alpha_2/\alpha_1|^2 > 1$. This result indicates that in this case, mode matching does not lead to the most efficient excitation scheme- a feature that resembles the notion of adjoint coupling in the context of free space unstable laser resonators [7].

6. Generalized eigenvectors of the resolvent

A direct consequence of resolvent definition is that it shares the same eigenvectors with its corresponding Hamiltonian, including the exceptional vector, i.e. $|K_1^r\rangle = |J_1^r\rangle$. However, the generalized eigenvectors of the Hamiltonian and those of the corresponding resolvent are not identical. Additionally, as we have mentioned before, $|K_n^r\rangle$ and $|J_n^r\rangle$ are not unique. However, once the two sets of vectors are chosen according to a specific criterion, one can

express the former in terms of the latter. In particular, one can write $|K_m^r\rangle = \sum_{i=1}^{M} C_i^m |J_i^r\rangle$, where the coefficients C_i^m are given by $C_i^m = \langle \tilde{J}_i^l | K_m^r\rangle$ (see supplementary note 2 for the discussion of the left vectors $\langle \tilde{J}_i^l|$).

In general, the above series expansion should be computed numerically. However, one particular solution can be expressed in closed form. In the following, we derive this result. Consider a choice of the vectors $|K_m^r\rangle$ as $|K_m^r\rangle = \sum_{i=2}^{m} C_i^m |J_i^r\rangle$, with $m = 2, 3, \ldots, M$. Note that the upper limit of the summation is m, not M, i.e. it varies from one vector to another. The strategy is to substitute this ansatz into the formula $(\hat{G}_{EP} - \mu_{EP}\hat{I})|K_m^r\rangle = \xi |K_{m-1}^r\rangle$ and solve for the vectors $|K_m^r\rangle$. Before we proceed, we recall $\mu_{EP} \equiv (\omega - \Omega_{EP})^{-1}$. Let us now consider the case $m = 2$. It follows that $(\hat{G}_{EP} - \mu_{EP}\hat{I})C_2^2 |J_2^r\rangle = \xi |J_1^r\rangle$. By multiplying both sides by $1/\hat{G}_{EP} \equiv (\omega\hat{I} - \hat{H}_{EP})$, we obtain $\mu_{EP} C_2^2 \chi_2 |J_1^r\rangle = \xi(\omega - \Omega_{EP})|J_1^r\rangle$, which in turn leads to:

$$C_2^2 = \frac{\xi}{\mu_{EP}^2 \chi_2}. \tag{6.1}$$

By following similar steps for $m > 2$, we arrive at the relation $\sum_{i=2}^{m} C_i^m \mu_{EP}\chi_i |J_{i-1}^r\rangle = \xi \sum_{i=2}^{m-1} \frac{C_i^{m-1}}{\mu_{EP}} |J_i^r\rangle - \xi \sum_{i=2}^{m-1} C_i^{m-1}\chi_i |J_{i-1}^r\rangle$. By changing the summation index $i \to i+1$ in the first and third terms, the above formula reads $\sum_{i=1}^{m-1} C_{i+1}^m \mu_{EP}\chi_{i+1} |J_i^r\rangle = \xi \sum_{i=2}^{m-1} \frac{C_i^{m-1}}{\mu_{EP}} |J_i^r\rangle - \xi \sum_{i=1}^{m-2} C_{i+1}^{m-1}\chi_{i+1} |J_i^r\rangle$. By rearranging the terms, we obtain $\left(C_2^m \mu_{EP}\chi_2 + \xi C_2^{m-1}\chi_2\right)|J_1^r\rangle + \sum_{i=2}^{m-2}\left(C_{i+1}^m \mu_{EP}\chi_{i+1} - \xi\frac{C_i^{m-1}}{\mu_{EP}} + \xi C_{i+1}^{m-1}\chi_{i+1}\right)|J_i^r\rangle + \left(C_m^m \mu_{EP}\chi_m - \xi\frac{C_{m-1}^{m-1}}{\mu_{EP}}\right)|J_{m-1}^r\rangle = 0$. By invoking the linear independence of vectors $|J_i^r\rangle$ and using the above expression for C_2^2 , we arrive at the following expressions for the coefficients C_2^m and C_m^m:

$$\begin{aligned} C_2^m &= \left(-\frac{1}{\mu_{EP}}\right)^m \frac{\xi^{m-1}}{\chi_2} \\ C_m^m &= \left(\frac{\xi}{\mu_{EP}^2}\right)^{m-1} \frac{1}{\chi_2\chi_3\cdots\chi_m} \end{aligned} \tag{6.2}$$

together with the recursive relation:

$$C_i^m = \left(\frac{1}{\mu_{EP}}\right)^2 \frac{\xi}{\chi_i} C_{i-1}^{m-1} - \frac{\xi}{\mu_{EP}} C_i^{m-1}, \tag{6.3}$$

where $i = 3, 4, \ldots, m-1$. Relation (6.3) is a recursive equation with variable coefficients. The unknown expansion constants C_i^m in Eq. (6.3) can be obtained by the aid of the known coefficients in Eq. (6.2). For instance, the value of C_3^4 depends on those of C_2^3 and C_3^3 which are obtained from the first and second lines of Eq. (6.2). In general, one can confirm by direct substitution that the solution of Eq. (6.3) can be written as:

$$C_i^m = (-1)^{m+i} \begin{pmatrix} m-2 \\ i-2 \end{pmatrix} \frac{\mu_{EP}^{2-m-i}}{\chi_2\chi_3\cdots\chi_i}\xi^{m-1}, \tag{6.4}$$

where $\begin{pmatrix} m \\ n \end{pmatrix} \equiv \frac{m!}{n!(m-n)!}$ is the binomial coefficient. Thus we finally arrive at:

$$|K_m^r\rangle = \sum_{i=2}^{m} (-1)^{m+i} \begin{pmatrix} m-2 \\ i-2 \end{pmatrix} \frac{\mu_{EP}^{2-m-i}\xi^{m-1}}{\chi_2\chi_3\cdots\chi_i} |J_i^r\rangle\,. \tag{6.5}$$

It is worth noting that the expansion coefficients obtained above are functions of the frequency ω of the input drive signal.

7. Systems with multiple exceptional points

Let us assume a Hamiltonian with two exceptional points of order, say, M_1 and M_2 and energy eigenvalues $\Omega_{EP}^{(1)}$, $\Omega_{EP}^{(2)}$ respectively (the case with several exceptional points is a straightforward generalization). The generalized eigenvalue equations for the left and right generalized eigenvectors associated with the two EPs are given by:

$$\left(\hat{H}_{EP} - \Omega_{EP}^{(1)}\right) |J_m^r\rangle = \chi_m^{(1)} |J_{m-1}^r\rangle \tag{7.1}$$

$$\langle J_m^l| \left(\hat{H}_{EP} - \Omega_{EP}^{(1)}\right) = \chi_m^{(1)} \langle J_{m-1}^l| \tag{7.2}$$

and

$$\left(\hat{H}_{EP} - \Omega_{EP}^{(2)}\right) |I_k^r\rangle = \chi_k^{(2)} |I_{k-1}^r\rangle \tag{7.3}$$

$$\langle I_k^l| \left(\hat{H}_{EP} - \Omega_{EP}^{(2)}\right) = \chi_k^{(2)} \langle I_{k-1}^l|\,, \tag{7.4}$$

where $m = 1, 2, \ldots, M_1$ and $k = 1, 2, \ldots, M_2$ and $\chi_1^{(1,2)} = 0$. In what follows, we show that the generalized eigenvectors associated with the two different EPs are biorthogonal. Let us start by inspecting the expression $\langle J_m^l| \left(\hat{H}_{EP} - \Omega_{EP}^{(1)}\right)^m |I_1^r\rangle$. It can be evaluated by first applying the operator $\left(\hat{H}_{EP} - \Omega_{EP}^{(1)}\right)^m$ to the vector $|I_1^r\rangle$ from the left or by acting on the vector $\langle J_m^l|$ from the right. By doing so, we obtain $\langle J_m^l|I_1^r\rangle = 0$. Similarly, one can show that $\langle J_1^l|I_k^r\rangle = 0$.

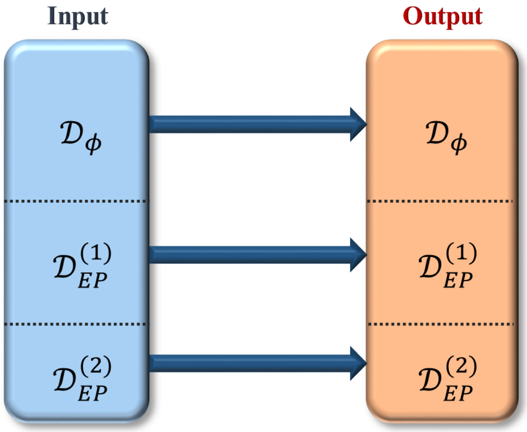

Supplementary Fig. 2. General structure of the linear response associated with a non-Hermitian system that exhibits two different EPs. As can be seen, each domain is coupled only to itself. In other words, there is no coupling between the two sub-spaces spanned by the generalized eigenvectors associated with the two EPs. Thick arrows indicate collective coupling between the domains. The details of the coupling (not shown here) follow a scheme identical to that depicted in Fig. 5**b**.

Next, we evaluate the expression $\langle J_m^l | \left(\hat{H}_{EP} - \Omega_{EP}^{(1)}\right) | I_k^r \rangle$ for $k, m > 1$. By following the procedure described above and by using Eqs. (7.2) and (7.3), we find $\left(\Omega_{EP}^{(2)} - \Omega_{EP}^{(1)}\right) \langle J_m^l | I_k^r \rangle = \chi_m^{(1)} \langle J_{m-1}^l | I_k^r \rangle - \chi_k^{(2)} \langle J_m^l | I_{k-1}^r \rangle$. The above formula can be used recursively to replace $\langle J_{m-1}^l | I_k^r \rangle$ and $\langle J_m^l | I_{k-1}^r \rangle$ by generalized eigenvectors having lower order indices to arrive at $\langle J_m^l | I_k^r \rangle = \sum_{m'=2}^{m} c_{m'}^{(1)} \langle J_{m'}^l | I_1^r \rangle + \sum_{k'=1}^{k} c_{k'}^{(2)} \langle J_1^l | I_{k'}^r \rangle$. But the right hand side of this last expression is zero, and hence $\langle J_m^l | I_k^r \rangle = 0$. Similarly, $\langle I_k^l | J_m^r \rangle = 0$. In other words, the generalized eigenvectors of the two different EPs form a biorthogonal set of vectors.

Equipped with this information, we can consider any arbitrary input signal $\sum_{n=1}^{N-M_1-M_2} c_n |\psi_n^r\rangle + \sum_{m=1}^{M_1} d_m |J_m^r\rangle + \sum_{k=1}^{M_2} f_k |I_k^r\rangle$, and repeat the same procedure as in supplementary note 4, to obtain:

$$\hat{G}_{EP} |u\rangle = \left(\sum_{n=1}^{N-M_1-M_2} \frac{|\psi_n^r\rangle \langle \psi_n^l|}{\omega - \Omega_n} + \sum_{m=1}^{M_1} \sum_{m'=m}^{M_1} \alpha_{m'}^{(m)} \frac{|J_m^r\rangle \langle \tilde{J}_{m'}^l|}{\left(\omega - \Omega_{EP}^{(1)}\right)^{m'-m+1}} + \sum_{k=1}^{M_2} \sum_{k'=k}^{M_2} \beta_{k'}^{(k)} \frac{|I_k^r\rangle \langle \tilde{I}_{k'}^l|}{\left(\omega - \Omega_{EP}^{(2)}\right)^{k'-k+1}} \right) |u\rangle \tag{7.5}$$

where $\alpha_m^{(m)} = 1$, $\alpha_{m'}^{(m)} = \alpha_{m'-1}^{(m)} \chi_{m'}^{(1)}$ and $\beta_k^{(k)} = 1$, $\beta_{k'}^{(k)} = \beta_{k'-1}^{(k)} \chi_{k'}^{(2)}$. As discussed in supple-

mentary note 2 for the case of only one EP, the vectors $\langle \tilde{J}^l_m|$ and $\langle \tilde{I}^l_k|$ span the dual domains associated with $\mathcal{D}^{(1,2)}_{EP}$ and satisfy the relations $\langle \tilde{J}^l_m|J^r_n\rangle = \delta_{n,m}$ and $\langle \tilde{I}^l_m|I^r_n\rangle = \delta_{n,m}$. The general structure described by the above expansion is shown schematically in supplementary Fig. 2.

8. Photonic simulations

Here we explain the details of the simulations associated with Fig. 6**a** in the main text. The outer radius and width of the microring resonator was chosen to be $R = 5$ µm and $w = 0.25$ µm, respectively. The material refractive index is assumed to be $n_r = 3.47$ and the background index was taken as $n_b = 1.44$ (relevant to silicon photonics). The straight waveguides are of the same material and width as the ring. The edge-to-edge separation between the ring and each of the waveguides is $d = 0.2$ µm. By first using an add-drop configuration (in the absence of the mirror) and plotting the drop signal as a function of frequency (see supplementary Fig. 3), we evaluate the value of the decay rate: $\gamma = 123\,\text{GHz}$. From the same figure, we also find that $\omega_o = 1217 \times 10^{12}\,\text{sec}^{-1}$, corresponding to $f_o = 193.7$ THZ or equivalently $\lambda_o = 1548$ nm for the TE optical mode. The mirror is implemented by using a 100-nm-thick silver layer. The absolute value of the amplitude reflection coefficient of the mirror is obtained by simulating the waveguide-mirror system in the absence of the ring resonator, and is found to be $|r| \approx 0.98$. The stored energy enhancement η is calculated by integrating the time averaged energy density over the ring resonator volume for resonant excitation from ports P_2 and P_1 under steady-state conditions (see Fig. 6**b** in the main text). All simulations were performed by using 2D finite element method available from COMSOL software package.

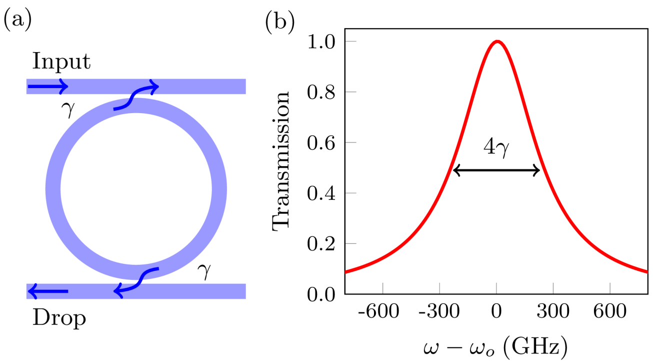

Supplementary Fig. 3. (a) Add-drop filter configuration for evaluating the resonant frequency and decay rate of a microring resonator. The design parameters of the ring resonator and the waveguides are listed in the text. (b) Optical power transmission as a function of frequency detuning from the resonant frequency (estimated from the simulation data to be $f_o = 193.7$ THZ) as obtained by frequency domain finite element numerical analysis. From the plot, we estimate that $\gamma = 123$ GHZ.

---

[1] M. G. Daly, P. E. Jessop, and D. Yevick, "Crosstalk reduction in intersecting rib waveguides," J. Lightw. Technol. **14**, 1695–1698 (1996).

[2] Sergei F. Mingaleev, Matthias Schillinger, Daniel Hermann, and Kurt Busch, "Tunable photonic crystal circuits: concepts and designs based on single-pore infiltration," Opt. Lett. **29**, 2858–2860 (2004).

[3] Yousuke Kobayashi and Hiroyuki Tsuda, "Crosstalk reduction using tapered intersecting waveguides," Opt. Rev. **12**, 387–390 (2005).

[4] Stefano Longhi, "Supersymmetric transparent optical intersections," Opt. Lett. **40**, 463–466 (2015).

[5] Q. Zhong, S.K. Özdemir, A. Eisfeld, A. Metelmann, and R. El-Ganainy, "Exceptional-point-based optical amplifiers," Phys. Rev. Applied **13**, 014070 (2020).

[6] A. E. Siegman, "Excess spontaneous emission in non-hermitian optical systems. i. laser amplifiers," Phys. Rev. A **39**, 1253–1263 (1989).

[7] A. E. Siegman, "Lasers without photons — or should it be lasers with too many photons?" Appl. Phys. B **60**, 247–257 (1995).
